# Prevalence of Geriatric Syndromes and the Need for Hospice Care in Older Patients of the Emergency Department: A Study in an Asian Medical Center

**DOI:** 10.1155/2020/7174695

**Published:** 2020-07-17

**Authors:** Ya-Ting Ke, An-Chi Peng, Yi-Min Shu, Min-Hsien Chung, Kang-Ting Tsai, Ping-Jen Chen, Tzu-Chieh Weng, Chien-Chin Hsu, Hung-Jung Lin, Chien-Cheng Huang

**Affiliations:** ^1^Department of Nursing, Chi Mei Medical Center, Tainan, Taiwan; ^2^Graduate Institute of Nursing, Kaohsiung Medical University, Kaohsiung City, Taiwan; ^3^Department of Senior Services, Southern Taiwan University of Science and Technology, Tainan, Taiwan; ^4^Department of Emergency Medicine, Kuo General Hospital, Tainan, Taiwan; ^5^Department of Geriatrics and Gerontology, Chi Mei Medical Center, Tainan, Taiwan; ^6^Department of Family Medicine, Kaohsiung Medical University, Kaohsiung, Taiwan; ^7^Holistic Care Unit, Department of Internal Medicine, Chi Mei Medical Center, Tainan, Taiwan; ^8^Department of Emergency Medicine, Chi Mei Medical Center, Tainan, Taiwan; ^9^Department of Biotechnology, Southern Taiwan University of Science and Technology, Tainan, Taiwan; ^10^Department of Emergency Medicine, Taipei Medical University, Taipei, Taiwan; ^11^Department of Environmental and Occupational Health, College of Medicine, National Cheng Kung University, Tainan, Taiwan

## Abstract

**Background:**

The prevalence of geriatric syndromes and the need for hospice care in the emergency department (ED) in Asian populations remain unclear. This study was conducted to fill the data gap.

**Methods:**

Using a newly developed emergency geriatric assessment (EGA), we investigated the prevalence of geriatric syndromes and the need for hospice care in older ED patients of a tertiary medical center between September 1, 2016, and January 31, 2017.

**Results:**

We recruited a total of 693 patients with a mean age of 78.0 years (standard deviation 8.2 years), comprising 46.6% of females. According to age subgroups, 37.4% of patients were aged 65–74 years, 37.4% were aged 75–84 years, and 25.2% were aged ≥85 years. The prevalence rates of geriatric syndromes were as follows: delirium (11.4%), depression (23.4%), dementia (43.1%), deterioration of activities of daily living (ADL) for <1 year (29.4%), vision impairment (22.2%), hearing impairment (23.8%), sleep disturbance (13.1%), any fall in <1 year (21.8%), polypharmacy (28.7%), pain (35.1%), pressure ulcer (5.6%), incontinence or retention (29.6%), indwelling device or physical restrain (21.6%), nutrition problem (35.7%), frequent use of medical resources (50.1%), lack of advance care planning (84.0%), caregiver problem (4.6%), socioeconomic problem (5.5%), and need for family meeting (6.2%). The need for hospice care was 11.9%. Most geriatric syndromes increased with advancing age except depression, sleep disturbance, polypharmacy, pain, nutrition problem, lack of advance care planning, caregiver problem, and socioeconomic problem.

**Conclusion:**

Geriatric syndromes and the need for hospice care were common in the older ED patients. Further studies about subsequent intervention for improving geriatric care are needed.

## 1. Introduction

A rapid increase in the older population is a common global phenomenon, especially in developed countries [1]. In the United States, the older population (aged ≥65 years) was 16.9% in 2020 and has been estimated to increase to 20.6% by the year 2030 [2]. Taiwan is one of the fast aging countries in the world [3]. Taiwan has become an “aged society” (people aged ≥65 years constituted 14%) in 2018 and is projected to become a “super-aged society” (people aged ≥65 years would constitute 20%) by 2025 [3]. Thus, aging has emerged as one of the most important issues in Taiwan [3].

The growing older population leads to increases in medical expenditures and challenges to the health care system. According to statistics from the Taiwan National Health Insurance, the older population contributed to 37% of total medical expenditures in 2017 and the percentage is still increasing [4]. Emergency departments (EDs) are the most common used medical resources when older people suffer from acute conditions [5–9]. A recent study in Taiwan reported that 35% of all ambulance calls for ED visits were made for the elderly population [8]. Older people also have a higher rate of revisiting the ED or readmission to the hospital than the younger population due to the complexity and multidimensionality of their problems and their comparative frailty [10–13]. Recent studies have demonstrated that an early comprehensive geriatric assessment (CGA) for identifying geriatric syndromes with subsequent intervention in the ED could reduce both admission and readmission rates [10–13]. However, the prevalence of geriatric syndromes and the need for hospice care in Asian older ED patients have not been investigated. Delineating the prevalence of geriatric syndromes and the need for hospice care would help the subsequent intervention for improving the quality of geriatric care in the ED. Therefore, we conducted this study in an ED in Taiwan with an aim of clarifying this issue and filling the data gap.

## 2. Materials and Methods

### 2.1. Study Design, Setting, and Participants

This study was conducted in the observation unit of the ED of the Chi Mei Medical Center (CMMC). The CMMC is a tertiary medical center in Southern Taiwan with an 80-bed ED staffed with board-certified emergency physicians who provide emergency care to approximately 145,000 patients per year [14]. Older patients (aged ≥65 years), who were waiting for admission in the ED, or being observed in the observation unit of the ED, were eligible for performing emergency geriatric assessment (EGA). Patients who were suffering from acute strokes and acute myocardial infarctions or were awaiting surgery were excluded. We recruited the patient if the treating physician or the nurse requested that the patient had the need for evaluation of geriatric syndromes or hospice care.

### 2.2. Variables and Data Collection

We used a recently published comprehensive assessment method, the “EGA,” for the assessment of older ED patients as a regular practice [15]. The EGA was developed based on the modified CGA with the addition of the need for hospice care and is intended to comprehensively screen older ED patients and initiate subsequent interventions to improve quality of care and reduce ED visits [15, 16]. The development processes of the EGA were as follows: (1) constructing draft EGA by literature review and committee, (2) modifying the EGA according to geriatric focus group comments, (3) revising the EGA by expert validation, (4) performing EGA in 118 older ED patients, and (5) assessing reliability and validity [15]. We provided a 3 h educational program for EGA, including on-hand practice to train the nurse practitioners or nurses. The EGA enables trained nurse practitioners or nurses to perform systematic and easy evaluation of patients. The following variables were included in the EGA: (1) delirium, (2) depression, (3) dementia, (4) deterioration of activities of daily living (ADL), (5) vision impairment affecting ADL, (6) hearing impairment affecting ADL, (7) sleep disturbances affecting ADL, (8) any fall in <1 year, (9) polypharmacy (≥8 drugs), (10) pain, (11) pressure ulcer, (12) incontinence or retention (urinary or fecal), (13) indwelling device or physical restrain, (14) nutrition problem, (15) frequent use of medical resources (≥2 ED visits or ≥2 admissions in 1 year), (16) lack of advance care planning (ACP), (17) caregiver problem, (18) socioeconomic problem, (19) need for family meeting and need for hospice care, (20) estimated life period <6 months, (21) need for comfort care only, and (22) long-term bedridden (i.e., coexistent long-term bedridden >3 months, recurrent infection, and significant cognitive impairment) [15, 16]. Age was categorized into the following three subgroups for the analysis: young elderly (age 65–74 years), moderately elderly (age 75–84 years), and old elderly (age ≥85 years) [17]. Delirium was assessed by the nurse using bCAM [15, 18]. Depression was assessed using three items, which was simplified from the Geriatric Depression Scale (GDS) as follows: (1) Did you feel unhappy in the past 2 weeks? (2) Do you prefer to stay at home rather than go out and do new things? (3) Do you feel worthless the way you are now? Depression was suspected if the patient has more than one positive item [15, 19]. Dementia was suspected by any problem recalling the following 3 items after completing 6^th^ item “Auditory”: bicycle, red, and happy [15]. ADL was assessed by 7 items as follows: walking, bathing, hygiene, toileting, dressing, transferring, and feeding. For the need and characteristics of our patients, the 7 items were modified from the Katz index and Barthel index after the multidisciplinary committee [15]. The drug count for polypharmacy included prescription drug only. The definition of “pain” included any complaint of pain without assessing intensity. Nutrition problem was defined as any of the following: (1) body mass index (BMI) ≤18.5 kg/m^2^, (2) BMI ≥27 kg/m^2^, (3) body weight loss (BWL) 5% in <1 month or BWL 10% in <6 months, (4) dysphagia or poor appetite in recent 2 weeks, or (5) difficulty in swallowing or intake [15]. Body weight was measured by seat scale or bed scale in the patients with bedridden status. The definition of the lack of ACP is the lack of any decision or discussion between patients and their relatives about their preferences and wishes for future care when they are at end-of-life. The definitions of the need for hospice care were made according to the following steps: (1) draft definitions constructed by literature review and expert committee based on the general criteria for end-of-life [20], (2) definitions modified according to geriatric focus group comments, (3) revision by expert validation, and (4) reliability and validity assessment in 118 geriatric ED patients [16]. Because there is no consensus for the time of end-of-life, we used “estimated life period <6 months” according to previous studies [21]. We retrospectively reviewed the results of the EGA between September 1, 2016, and January 31, 2017, collected by a trained nurse practitioner who was blind to the outcomes of the recruited patients.

### 2.3. Statistical Methods

The independent *t*-test or the Mann–Whitney–Wilcoxon test was used for assessing continuous variables, and Pearson's chi-square test or Fisher's exact test was used for assessing categorical variables to compare between female and male patients. We used the Cochran–Armitage test for trend to compare the difference among three age subgroups. The significance level was set at 0.05 (two-tailed). All statistical analyses were conducted using IBM SPSS Statistics 20.0.

### 2.4. Ethical Statements

This study was approved by the institutional review board (IRB) at the Chi Mei Medical Center. The IRB waived the need for informed consent from the patients as this was a retrospective study. All analyzed data were anonymized.

## 3. Results

A total of 693 older patients (323 females and 370 males) were recruited into this study after excluding 19 patients with incomplete data (Table 1). The mean age of the patients was 78.0 years (standard deviation 8.2 years), with no sex difference. Regarding age subgroups, there were 37.4% of patients in each of the 65–74-year and the 75–84-year subgroups, and 25.2% of patients were aged ≥85 years. The mean time between ED visit and admission was about 4.8 hours. Most of the information was collected from the patients themselves (72.9%) and their families (62.3%). The amount of information collected from families was higher in female patients than in male patients (73.4% vs. 65.7%, *p*=0.04).

The prevalence of delirium, depression, and dementia was 11.4%, 23.4%, and 43.1%, with no sex difference, respectively (Table 2). Regarding ADL, 29.4% of the patients had a deterioration of ADL for <1 year, and the subtypes of deteriorated ADL included walking (20.5%), bathing (20.2%), hygiene (19.2%), toileting (16.0%), dressing (15.3%), transferring (13.3%), and feeding (3.3%). In addition, 22.2% of the patients had vision impairment affecting ADL. Hearing impairment affecting ADL was present in 23.8% of patients and was higher in male than female patients. Female patients had a higher prevalence of sleep disturbances affecting ADL than male patients (15.9% vs. 10.7%, *p*=0.04). The prevalence rates of any fall in <1 year and polypharmacy were 21.8% and 28.7% with no sex difference. One-third of the patients complained of pain, and female patients showed a trend of higher prevalence of pain than male patients. The problem of urinary or fecal incontinence or retention was observed in 29.6% of the patients. Indwelling device or physical restrain was found in 21.6% of the patients, and the most common indwelling devices were Foley catheter (14.2%) and nasogastric tube (11.7%). The prevalence of nutrition problems was higher in female patients than in male patients (43.3% vs. 29.0%). There was no difference in BMI between the two sexes. Half of the patients had the problem of frequent use of medical resources. Up to 84.0% of patients did not have preparation for ACP, and 4.6% and 5.5% of the patients had caregiver problems and socioeconomic problems, respectively. The issue of living alone was found in 4.5% of the patients. Family meetings were required in 6.2% of the patients for discussion about the subsequent care plan. In total, 11.9% of the patients were found to need hospice care, and the most common criteria fulfilled were being long-term bedridden for >3 months, recurrent infection, and significant cognitive impairment.

In the comparison among three age subgroups, the prevalence of delirium, dementia, deterioration of ADL <1 year, vision impairment affecting ADL, hearing impairment affecting ADL, any fall <1 year, pressure ulcer, incontinence or retention, indwelling device or physical restrain, frequent use of medical resource, need for family meeting, and need for hospice care increased with advancing age (Table 3). However, with advancing age, there was a decreasing trend of prevalence of sleep disturbances affecting ADL, lack of ACP, and caregiver problem. The difference of prevalence rate of depression, polypharmacy, pain, nutrition problem, and socioeconomic problem was not significant among three age subgroups.

## 4. Discussion

This study revealed that patients aged ≥85 years constituted 25.2% of the older ED patients, which reminds us of the heavy medical burden imposed by an aging society. In the literature search, we did not find data about this issue in Taiwan; however, a nationwide study in Taiwan reported that 11.6% of older patients with trauma were aged ≥85 years in 2007 [22]. Another study in Canada conducted in 2014 reported that 18.2% of older ED patients were aged ≥85 years [23]. In Taiwan, the older population constituted 10.2% in 2007 and rapidly increased to 14% in 2018 [3], and therefore the percentage of old elderly in the ED is projected to be increasing in the future.

In the present study, a high proportion of patients was found to have no preparation for ACP and patients aged ≥85 years had higher preparation for ACP than younger patients. Similarly, an earlier study of an ED in Australia conducted in 2015 had reported that 86.7% of older patients did not have ACP [24]. ACP has been recognized as a tool for facilitating decision-making in the ED and reducing hospitalization in the older population through the early identification of patients' needs and wishes, as well as better future planning [24–26]. ACP allows patients and their caregivers to achieve a shared understanding of goals and preferences for the future, including refusal to receive certain treatments or interventions, and nominates a power of attorney to indicate the wishes of the patients should they lose their mental capacity [26]. The ACP system in Taiwan, named “Patient Right to Autonomy Act,” has took effect in 2019 [27].

In this study, half of the patients were found to have the problem of frequent use of medical resources, which has been recognized as a feature of frail older people [11]. The suggested solution to this problem is detailed, multidimensional, and interdisciplinary care, which could provide the correct diagnosis, management, and appropriate ongoing care or avoid admission [11]. In this study, 43.1% of the patients fulfilled the screening criteria for dementia. This is a very high proportion compared to the 8.04% prevalence of dementia in the general older population in Taiwan [28]. Although this figure is only a screening data and not a definite diagnosis of dementia, it still reflects the fact that older patients with dementia are high users of medical resources [29]. Validation of the screening criteria used in this study in the future is warranted.

Nutrition problem was found in 35.7% of the patients in this study based on the screening criteria. Patients aged ≥85 years had the highest prevalence of nutrition problem, followed by patients aged 75–84 years and 65–74 years. However, the difference among three age subgroups was not significant. A 2015 study conducted in the United States using the Mini Nutritional Assessment Short-Form as a screener reported that 16% of older ED patients were malnourished and 60% were either malnourished or at risk of malnutrition [30]. Malnutrition is a common and long-standing problem among the older population and is often unrecognized. It is also associated with increased use of medical resources and negative outcomes, including increased mortality, complications, and length of hospital stay [31]. Increased BMI and overweight are beneficial to older adults [32]. It has been reported that after performing nutrition screening and assessment, interventions including dietary modifications, enteral or parenteral nutrition, feeding assistance, and swallowing rehabilitation are necessary [31, 33].

In the present study, pain was present in 35.1% of the patients and the prevalence of pain did not increase with advancing age. A study conducted in Europe reported that 42.0% of the older population have chronic pain [34], and another study reported that pain was even more common in older hospitalized patients with a prevalence of 67.3% [35]. Pain management is extremely important because pain may induce several complications, including depression, deconditioning, impaired ADL, cognitive decline, accidents, and polypharmacy [36]. The reason that the prevalence of pain did not increase with advancing age may be due to more impaired cognition and misinterpretation of pain as a normal aging response in the old elderly, which affect the diagnosis of pain [36].

Urinary incontinence and fecal incontinence (or retention) are common, distressing, and often disabling conditions in the older people. These two problems may occur together because of similar etiologies and risk factors [37]. Although incontinence is not fatal, it causes embarrassment, decreased quality of life, care burdens, and complications such as infection and pressure ulcers [37].

Deterioration of ADL indicates a functional impairment, which if not corrected can lead to loss of independence and risk of institutionalization [11]. In addition, the decline in ADL suggests a presentation of an underlying disease, which would explain its association with a higher mortality rate [11]. Falling is one of the most common causes of a geriatric visit to the ED [11]. It is also closely associated with frailty in the older people and serves as an indicator for further referral through a multidisciplinary review [11]. Immediate assessment of the falls is suggested in the ED, and referral to specialist multifactorial interventions is essential [11]. Older people find it difficult to avoid polypharmacy because of the multiple comorbidities. However, it still should be emphasized due to the direct relationship between the number of drugs and the drug interactions [11]. A recent study showed that early initiation of computer-based and pharmacist-assisted intervention in the ED could reduce major polypharmacy and potentially inappropriate medication [38]. Previous research has reported that 14%–20% of the community older adults experience depressive symptoms, with higher rates among the hospitalized older people (12%–45%) and even higher rates among the older population living in long-term care facilities (an estimated 40%) [39]. In contrast to young people, older adults with depression often have multiple concurrent comorbidities and suffer from cognitive impairment [40]. Depression is often undetected or inadequately treated in the older population because of a lack of awareness among health care providers or caregivers [40]. Depression in the older adults requires more attention because it increases the functional decline, which may result in placement in a facility, family stress, a higher risk for other illnesses, reduced recovery from illness, and premature death due to suicide and other causes [40]. Past research has reported that 11%–24% of hospitalized older patients suffer from delirium [11]. Early detection of delirium in the ED is crucial as it is associated with underlying diseases and a subsequent increased length of hospital stay, admission to residential care, and death [11]. There is a diagnostic difficulty for differencing delirium and dementia because the interrelation between them remains poorly understood [41]. Many hypotheses are proposed as follows: (1) delirium is a marker of vulnerability to dementia; (2) delirium is related to its precipitating factors; and (3) delirium may cause permanent neuronal damage and lead to dementia [41]. The present study showed that hearing impairment was more common in female than in male patients, a result that is comparable to that of previous studies [42]. Hearing impairment is common in the older population and has been reported to result in communication problems, social isolation, disability, decreased quality of life, and increased numbers of accidents [11]. The higher prevalence of sleep disturbances in female compared to male patients in this study is also comparable with the findings of previous research [43]. A recent study conducted in Korea reported that 56.4% of female older adults had poor sleep, which was significantly higher than 38.6% of male counterparts [43]. Sleep disturbances can result in decreased quality of life, increased psychiatric disorders, inappropriate use of sleep aids, and decreased ADL [43, 44]. In this study, patients aged ≥85 years had the lowest prevalence of sleep disturbances, followed by patients aged 75–84 years and patients aged 65–74 years. A large study in the United States reported that advancing age was not associated with increased self-reported sleep disturbance, which is compatible with our finding [45]. The possible explanation is that sleep disturbances with age is a nonlinear phenomenon and medicated by factors other than physiologic aging [45].

The present study showed that 11.9% of patients had the need for hospice care. Among 82 patients, all had the reason “long-term bedridden for >3 months, recurrent infection, and significant cognitive impairment” for the need for hospice care. The other reasons were “estimated life period <6 months (26 patients)” and “need for comfortable care only (26 patients).” A recent study in our institution demonstrated that 74.1% of patients who received hospice and palliative care in the ED were the older population [16]. Identifying the needs for hospice and palliative care can prove to be pivotal to preventing admission with anticipatory care or prioritizing the patient's wishes [11].

National guideline suggests performing CGA for frail older patients admitted to the hospitals because it is beneficial for subsequent prognosis [46]. However, there is no clear evidence that CGA conducted in the ED (e.g., EGA in this study) reduced reattendance and readmission [46]. Further research about screening for target populations and increased intensity of interventions is needed to clarify this issue [46].

Although the strength of this study is that it is the first to evaluate the prevalence of geriatric syndromes and the need for hospice care in an Asian ED, it still has some limitations. First, this is a descriptive study where the conclusions are based essentially only on correlations. Second, we used an EGA developed by our institution, which has not been validated in other hospitals or nations, as the screening method. Nevertheless, we have attempted our best to clarify this issue because there is still no consensus about how to perform comprehensive assessment in the older ED patients. Third, we recruited the study patients in a convenient manner, which may not reflect the actual population. The total potential sample and proportion of patients who did not have EGA were not calculated. There was also the possibility of day/week and time/day bias in the administration of the EGA. As our institution is a tertiary medical center, the recruited patients may be in more critical states than those in other hospitals. Fourth, we did not investigate the targeted interventions and the proportions of avoidable acute admissions and those requiring hospice care who died in the hospital or were discharged elsewhere. Because our aim was to clarify the prevalence of geriatric syndromes and the need for hospice care in the ED, evaluation of targeted interventions, avoidable acute admissions, and follow-up of the patients will be performed in a subsequent study. Fifth, we did not collect the data of patient's residence, including community dwelling and long-term care facility, which may be beneficial for subsequent analyses. Sixth, the definition of “nutrition problem” may not be appropriate because malnutrition is a risk factor of poor prognosis while overweight is often considered as a protective factor in older age, especially in the oldest old. Therefore, dividing “nutrition problem” into “malnutrition” and “overweight” may be a better choice. Further studies validating the methods that we adopted, recruiting patients in a more comprehensive manner, and investigating patients in other hospitals in Taiwan or other Asian nations are warranted.

## 5. Conclusions

This study delineated the prevalence of geriatric syndromes and the need for hospice care in an Asian ED. Up to one-fourth of the older ED patients were aged ≥85 years. Geriatric syndromes were common, especially the lack of ACP, frequent use of medical resources, dementia, nutrition problem, pain, incontinence or retention, deterioration of ADL for <1 year, polypharmacy, and depression. Delirium was detected in 11.4% of the patients. Hospice care was required by 11.9% of the patients. Most geriatric syndromes increased with advancing age. The results of this study provide a general picture of the geriatric syndromes and the need for hospice care in an Asian ED, which could facilitate the adoption of associated referrals and interventions as early as possible and improve future geriatric care. Further studies validating the methods adopted in this study in other Asian hospitals and subsequent assessments and improvements of the results are warranted.

## Figures and Tables

**Table 1:** Age, sex, and data sources of the participants.

Variable	Total (*n* = 693)	Female (*n* = 323)	Male (*n* = 370)	*p* value^*∗*^
Age (years)	78.0 ± 8.2	78.0 ± 8.5	78.0 ± 8.1	0.97
Age subgroup				0.79
65–74 years	259 (37.4)	125 (38.7)	134 (36.2)	
75–84 years	259 (37.4)	119 (36.8)	140 (37.8)	
≥85 years	175 (25.2)	79 (24.5)	96 (25.9)	
Data sources^†^				
Patient	505 (72.9)	241 (74.6)	264 (71.4)	0.41
Family	480 (62.3)	237 (73.4)	243 (65.7)	0.04
Caregiver	79 (11.4)	35 (10.8)	44 (11.9)	0.74
Others	2 (0.3)	1 (0.3)	1 (0.3)	>0.95

Data are presented as mean ± standard deviation or *n* (%). ^*∗*^Comparison between female and male participants. ^†^Data sources may be multiple.

**Table 2:** Prevalence of geriatric syndromes and the need for hospice care based on EGA classified by sex.

Variable	Total (*n* = 693)	Female (*n* = 323)	Male (*n* = 370)	*p* value^*∗*^
Delirium	73 (11.4)	28 (9.4)	45 (13.1)	0.18
Depression	133 (23.4)	56 (21.5)	77 (25.1)	0.36
Dementia	248 (43.1)	123 (45.7)	125 (40.8)	0.27
Deterioration of ADL for <1 year^†^	200 (29.4)	97 (30.5)	103 (28.4)	0.60
Feeding	92 (3.3)	45 (13.9)	47 (12.7)	0.73
Hygiene	133 (19.2)	62 (19.2)	71 (19.2)	>0.95
Dressing	106 (15.3)	49 (15.2)	57 (15.4)	>0.95
Transferring	92 (13.3)	42 (13.0)	50 (13.6)	0.92
Walking	142 (20.5)	68 (21.1)	74 (20.1)	0.82
Toileting	111 (16.0)	53 (16.4)	58 (15.7)	0.89
Bathing	140 (20.2)	71 (22.0)	69 (18.7)	0.33
Vision impairment affecting ADL	143 (22.2)	66 (21.9)	77 (22.6)	0.90
Hearing impairment affecting ADL	153 (23.8)	59 (19.6)	94 (27.6)	0.02
Sleep disturbances affecting ADL	90 (13.1)	51 (15.9)	39 (10.7)	0.04
Any fall in <1 year	149 (21.8)	77 (24.1)	72 (19.7)	0.19
Polypharmacy (≥8 drugs)	198 (28.7)	98 (30.5)	100 (27.2)	0.38
Pain	220 (35.1)	114 (39.2)	106 (31.5)	0.06
Pressure ulcer	39 (5.6)	16 (5.0)	23 (6.2)	0.57
Incontinence or retention	205 (29.6)	96 (29.7)	109 (29.5)	>0.95
Indwelling device or physical restrain^†^	149 (21.6)	65 (20.2)	84 (22.8)	0.47
Nasogastric tube	81 (11.7)	31 (9.6)	50 (13.6)	0.14
Foley catheter	98 (14.2)	51 (15.8)	47 (12.7)	0.30
Tracheostomy	6 (0.9)	1 (0.3)	5 (1.4)	0.29
Physical restrain	5 (0.7)	3 (0.9)	2 (0.5)	0.88
Other	14 (2.0)	2 (0.6)	12 (3.3)	0.03
Nutrition problem^‡^	237 (35.7)	135 (43.3)	102 (29.0)	<0.01
BMI	22.9 ± 4.1	23.2 ± 4.6	22.7 ± 3.6	0.13
Frequent use of medical resources	347 (50.1)	150 (46.4)	197 (53.4)	0.08
Lack of advance care planning	581 (84.0)	269 (83.3)	312 (84.6)	0.73
Caregiver problem	32 (4.6)	11 (3.4)	21 (5.7)	0.21
Socioeconomic problem^†^	38 (5.5)	20 (6.7)	18 (4.9)	0.55
Live alone	31 (4.5)	17 (5.3)	14 (3.8)	0.46
Economic problem	10 (1.4)	4 (1.2)	6 (1.6)	0.92
Need for family meeting	43 (6.2)	19 (5.9)	24 (6.5)	0.87
Need for hospice care	82 (11.9)	38 (11.8)	44 (11.9)	>0.95
Estimated life period <6 months	26 (3.8)	14 (4.3)	12 (3.3)	0.59
Need for comfortable care only	26 (3.8)	14 (4.3)	12 (3.3)	0.59
Long-term bedridden^¶^	82 (11.9)	38 (11.9)	44 (11.9)	>0.95

Data are presented as mean ± standard deviation or *n* (%). ^*∗*^Comparison between female and male patients. ^†^Multiple choices. EGA, emergency geriatric assessment; ADL, activities of daily living; BMI, body mass index; BWL, body weight loss. ^‡^Any of the following: BMI ≤18.5 kg/m^2^, BMI ≥27 kg/m^2^, BWL 5% in <1 month or BWL 10% in <6 months, dysphagia or poor appetite in recent 2 weeks, or difficulty in swallowing or intake. ^¶^Long-term bedridden for >3 months, recurrent infection, and significant cognitive impairment.

**Table 3:** Prevalence of geriatric syndromes and needs for hospice care based on EGA classified by three age groups.

Variable	Total (*n* = 693)	Young elderly, 65–74 years (*n* = 259)	Moderately elderly, 75–84 years (*n* = 259)	Old elderly, ≥85 years (*n* = 175)	*p* value^*∗*^	*p* value for trend
Delirium	73 (11.4)	12 (4.8)	24 (10.0)	37 (24.8)	<0.01	<0.01
Depression	133 (23.4)	53 (22.1)	48 (22.2)	32 (28.6)	0.36	0.24
Dementia	248 (43.1)	61 (25.4)	112 (51.4)	75 (64.1)	<0.01	<0.01
Deterioration of ADL <1 year^†^	200 (29.4)	45 (17.8)	76 (29.6)	79 (46.2)	<0.01	<0.01
Feeding	92 (13.3)	11 (4.3)	30 (11.6)	51 (29.3)	<0.01	<0.01
Hygiene	133 (19.2)	26 (10.0)	50 (19.3)	57 (32.8)	<0.01	<0.01
Dressing	106 (15.3)	18 (7.0)	32 (12.4)	56 (32.2)	<0.01	<0.01
Transferring	92 (13.3)	12 (4.6)	32 (12.4)	48 (27.6)	<0.01	<0.01
Walking	142 (20.5)	29 (11.2)	55 (21.2)	58 (33.3)	<0.01	<0.01
Toileting	111 (16.0)	15 (5.8)	38 (14.7)	58 (33.3)	<0.01	<0.01
Bathing	140 (20.2)	25 (9.7)	50 (19.3)	65 (37.4)	<0.01	<0.01
Vision impairment affecting ADL	143 (22.2)	35 (13.8)	50 (20.8)	58 (38.9)	<0.01	<0.01
Hearing impairment affecting ADL	153 (23.8)	28 (11.0)	59 (24.7)	66 (44.3)	<0.01	<0.01
Sleep disturbances affecting ADL	90 (13.1)	45 (17.5)	26 (10.1)	19 (11.1)	0.03	0.03
Any fall <1 year	149 (21.8)	40 (15.6)	62 (24.1)	47 (27.5)	<0.01	<0.01
Polypharmacy (≥8 drugs)	198 (28.7)	81 (31.4)	66 (25.5)	51 (29.7)	0.32	0.57
Pain	220 (35.1)	84 (33.7)	84 (36.1)	52 (35.9)	0.85	0.63
Pressure ulcer	39 (5.6)	6 (2.3)	15 (5.8)	18 (10.3)	<0.01	<0.01
Incontinence or retention	205 (29.6)	39 (15.1)	78 (30.1)	88 (50.6)	<0.01	<0.01
Indwelling device or physical restrain^†^	149 (21.6)	36 (13.9)	59 (22.8)	54 (31.2)	<0.01	<0.01
Nasogastric tube	81 (11.7)	19 (7.3)	32 (12.4)	30 (17.2)	<0.01	<0.01
Foley	98 (14.2)	18 (7.0)	41 (15.8)	39 (22.4)	<0.01	<0.01
Tracheostomy	6 (0.9)	3 (1.2)	1 (0.4)	2 (1.2)	0.66	0.89
Physical restrain	5 (0.7)	0 (0.0)	0 (0.0)	5 (2.9)	<0.01	<0.01
Other	14 (2.0)	7 (2.7)	6 (2.3)	1 (0.6)	0.28	0.14
Nutrition problem^‡^	237 (35.7)	86 (34.0)	86 (34.4)	65 (40.4)	0.36	0.22
BMI	22.9 ± 4.1	23.8 ± 4.3	22.6 ± 3.9	21.9 ± 3.8	<0.01	<0.01
Frequent use of medical resource	347 (50.1)	120 (46.3)	126 (48.7)	101 (58.1)	<0.05	0.02
Lack of advance care planning	581 (84.0)	227 (87.6)	227 (87.6)	127 (73.0)	<0.01	<0.01
Caregiver problem	32 (4.6)	19 (7.3)	10 (3.9)	3 (1.7)	0.02	<0.01
Socioeconomic problem†	38 (5.5)	16 (6.2)	15 (5.8)	7 (4.1)	0.61	0.36
Live alone	31 (4.5)	13 (5.0)	13 (5.0)	5 (2.9)	0.50	0.32
Economic problem	10 (1.5)	5 (1.9)	3 (1.2)	2 (1.2)	0.72	0.47
Need for family meeting	43 (6.2)	9 (3.5)	14 (5.4)	20 (11.5)	<0.01	<0.01
Need for hospice care	82 (11.9)	16 (6.2)	31 (12.1)	35 (20.2)	<0.01	<0.01
Estimated life period <6 months	26 (3.8)	7 (2.7)	6 (2.3)	13 (7.5)	0.01	0.02
Need for comfortable care only	26 (3.8)	7 (2.7)	8 (3.1)	11 (6.3)	0.12	0.07
Long-term bedridden^¶^	82 (11.9)	15 (5.8)	31 (12.0)	36 (20.7)	<0.01	<0.01

Data are presented as mean ± standard deviation or *n* (%). EGA, emergency geriatric assessment; ADL, activities of daily living; BMI, body mass index. ^*∗*^Comparison among three age groups. ^†^Multiple choices. ^‡^Any of the following: BMI ≤18.5, BMI ≥27, BWL 5% <1 month or BWL 10% <6 months, dysphagia or poor appetite in recent 2 weeks, or difficulty of swallowing or intake. ^¶^Long-term bedridden >3 months, recurrent infection, and significant cognitive impairment.

## Data Availability

The datasets used and/or analyzed during the current study are available from the corresponding author upon reasonable request.

## References

[1] WHO *The Global Strategy and Action Plan on Ageing and Health*.

[2] USC Bureau (2017). *National Population Projections Tables*.

[3] TN. Statistics, Statistics Report, http://www.stat.gov.tw/public/Data/51021161625L3E5JF46.pdf

[4] Ministry of Health and Welfare, Statistics of National Health Insurance, Ministry of Health and Welfare, Washington, DC, USA, https://www.mohw.gov.tw/dl-50575-c76c27ab-715e-4ca5-af1c-008d848cdcb4.html

[5] Roberts D. C., McKay M. P., Shaffer A. (2008). Increasing rates of emergency department visits for elderly patients in the United States, 1993 to 2003. *Annals of Emergency Medicine*.

[6] Samaras N., Chevalley T., Samaras D., Gold G. (2010). Older patients in the emergency department: a review. *Annals of Emergency Medicine*.

[7] Liao M. C. C., Chou L. K., Laing M. Y. (2012). Effectiveness of comprehensive geriatric assessment-based intervention to reduce frequent emergency department visits: a report of four cases. *International Journal of Gerontology*.

[8] Huang C. C. C., Hsu W. L., Lin C. C. (2016). Elderly and non-elderly use of a dedicated ambulance corps’ emergency medical services in Taiwan. *BioMed Research International*.

[9] Huang H. S., Hsu C. C., Ye J. C., Su S. B., Huang C. C., Lin H. J. (2017). Predicting the mortality in geriatric patients with dengue fever. *Medicine (Baltimore)*.

[10] Conroy S. P., Ansari K., Williams M. (2014). A controlled evaluation of comprehensive geriatric assessment in the emergency department: the “emergency frailty unit”. *Age Ageing*.

[11] Ellis G., Marshall T., Ritchie C. (2014). Comprehensive geriatric assessment in the emergency department. *Clinical Interventions in Aging*.

[12] Ahmed N., Taylor K., McDaniel Y., Dyer C. B. (2012). The role of an acute care for the elderly unit in achieving hospital quality indicators while caring for frail hospitalized elders. *Population Health Management*.

[13] Palmer R. M. (2018). The acute care for elders unit model of care. *Geriatrics (Basel)*.

[14] Huang C. C., Hsu C. C., Guo H. R., Su S. B., Lin H. J. (2017). Dengue fever mortality score: a novel decision rule to predict death from dengue fever. *Journal of Infection*.

[15] Ke Y. T., Peng A. C., Shu Y. M. (2018). Emergency geriatric assessment: a novel comprehensive screen tool for geriatric patients in the emergency department. *The American Journal of Emergency Medicine*.

[16] Weng T. C., Yang Y. C., Chen P. J. (2017). Implementing a novel model for hospice and palliative care in the emergency department: an experience from a tertiary medical center in Taiwan. *Medicine (Baltimore)*.

[17] Chung M. H., Huang C. C., Vong S. C. (2014). Geriatric fever score: a new decision rule for geriatric care. *PLoS One*.

[18] Baten V., Busch H. J., Busche C. (2018). Validation of the brief confusion assessment method for screening delirium in elderly medical patients in a German emergency department. *Academic Emergency Medicine*.

[19] Rinaldi P., Mecocci P., Benedetti C. (2003). Validation of the five-item geriatric depression scale in elderly subjects in three different settings. *Journal of the American Geriatrics Society*.

[20] Hui D., De La Cruz M., Mori M. (2013). Concepts and definitions for “supportive care,” “best supportive care,” “palliative care,” and “hospice care” in the published literature, dictionaries, and textbooks. *Support Care Cancer*.

[21] Batchelor N. H. (2010). Palliative or hospice care? Understanding the similarities and differences. *Rehabil Nurs*.

[22] Chou C. H., Pai C. H., Kao S., Lai L., Lai C. H., Chien W. C. (2011). The injury types and medical expenditure of Taiwanese elderly patients admitted to emergency department in 2007. *Taiwan Geriatr Gerontol*.

[23] Latham L. P., Ackroyd-Stolarz S. (2014). Emergency department utilization by older adults: a descriptive study. *Canadian Geriatrics Journal*.

[24] Street M., Ottmann G., Johnstone M. J., Considine J., Livingston P. M. (2015). Advance care planning for older people in Australia presenting to the emergency department from the community or residential aged care facilities. *Health & Social Care in the Community*.

[25] Platts-Mills T. F., Richmond N. L., LeFebvre E. M. (2017). Availability of advance care planning documentation for older emergency department patients: a cross-sectional study. *Journal of Palliative Medicine*.

[26] Robinson L., Dickinson C., Rousseau N. (2012). A systematic review of the effectiveness of advance care planning interventions for people with cognitive impairment and dementia. *Age Ageing*.

[27] M. o.H. a. Welfare, Patient Right to Autonomy Act, https://law.moj.gov.tw/ENG/LawClass/LawAll.aspx?pcode=L0020189

[28] Sun Y., Lee H.-J., Yang S.-C. (2014). A nationwide survey of mild cognitive impairment and dementia, including very mild dementia, in Taiwan. *PLoS One*.

[29] LaMantia M. A., Stump T. E., Messina F. C., Miller D. K., Callahan C. M. (2016). Emergency department use among older adults with dementia. *Alzheimer Disease & Associated Disorders*.

[30] Pereira G. F., Bulik C. M., Weaver M. A., Holland W. C., Platts-Mills T. F. (2015). Malnutrition among cognitively intact, noncritically ill older adults in the emergency department. *Annals of Emergency Medicine*.

[31] Avelino-Silva T. J., Jaluul O. (2017). Malnutrition in hospitalized older patients: management strategies to improve patient care and clinical outcomes. *International Journal of Gerontology*.

[32] Gill L. E., Bartels S. J., Batsis J. A. (2015). Weight management in older adults. *Current Obesity Reports*.

[33] Sura L., Madhavan A., Carnaby G., Crary M. A. (2012). Dysphagia in the elderly: management and nutritional considerations. *Clinical Interventions in Aging*.

[34] Kozak-Szkopek E., Broczek K., Slusarczyk P. (2017). Prevalence of chronic pain in the elderly Polish population—results of the PolSenior study. *Archives of Medical Science*.

[35] Gianni W., Madaio R. A., Di Cioccio L. (2010). Prevalence of pain in elderly hospitalized patients. *Archives of Gerontology and Geriatrics*.

[36] Kaye A. D., Baluch A., Scott J. T. (2010). Pain management in the elderly population: a review. *Ochsner Journal*.

[37] Tong Y. C. (2007). Global view on urinary and fecal incontinence. *Incontent Pelvic Floor Dysfunction*.

[38] Liu Y.-L., Chu L.-L., Su H.-C. (2019). Impact of computer-based and pharmacist-assisted medication review initiated in the emergency department. *Journal of the American Geriatrics Society*.

[39] Wiese B. S. (2011). Geriatric depression: the use of antidepressants in the elderly. *BCMJ*.

[40] Kok R. M., Reynolds C. F. (2017). Management of depression in older adults: a review. *JAMA*.

[41] Fong T. G., Davis D., Growdon M. E., Albuquerque A., Inouye S. K. (2015). The interface between delirium and dementia in elderly adults. *The Lancet Neurology*.

[42] Pearson J. D., Morrell C. H., Gordon-Salant S. (1995). Gender differences in a longitudinal study of age-associated hearing loss. *The Journal of the Acoustical Society of America*.

[43] Quan S.-A., Li Y.-C., Li W.-J., Li Y., Jeong J.-Y., Kim D.-H. (2016). Gender differences in sleep disturbance among elderly Koreans: hallym aging study. *Journal of Korean Medical Science*.

[44] Harrington J. J., Avidan A. Y. (2005). Treatment of sleep disorders in elderly patients. *Current Treatment Options in Neurology*.

[45] Grandner M. A., Martin J. L., Patel N. P. (2012). Age and sleep disturbances among American men and women: data from the U.S. behavioral risk factor surveillance system. *Sleep*.

[46] Harding S. (2020). Comprehensive geriatric assessment in the emergency department. *Age Ageing*.

